# Epoxy Blends Containing Melamine Phosphate-Based Flame Retardants: Thermal and Flammability Performance

**DOI:** 10.3390/ma19132877

**Published:** 2026-07-05

**Authors:** Magdalena Rogulska, Bogdan Tarasiuk, Przemysław Rybiński, Beata Podkościelna

**Affiliations:** 1 Department of Polymer Chemistry, Institute of Chemical Sciences, Faculty of Chemistry, Maria Curie-Skłodowska University, Gliniana St. 33, 20-614 Lublin, Poland; mrogulska@umcs.lublin.pl (M.R.); bogtar2020@wp.pl (B.T.); 2 Institute of Chemistry, Jan Kochanowski University, Uniwersytecka St. 7, 25-406 Kielce, Poland; przemyslaw.rybinski@ujk.edu.pl

**Keywords:** epoxy resin, flame retardants, DSC, thermogravimetric analysis, flammability test

## Abstract

Epoxy resins are widely used in advanced engineering applications, including coatings, adhesives, and electronics. Therefore, improving their flame resistance is important for enhancing fire safety and extending their range of applications. A series of flame retardants based on melamine phosphate derivatives, such as melamine phosphate (MP), melamine dibutyl phosphate, and melamine bis(2-ethylhexyl) phosphate, as well as a zinc borate-modified system (ZnB-MP) has been incorporated into commercially available epoxy resin (Epidian® 601). The blends were characterized using Fourier transform infrared spectroscopy (FTIR) to confirm their chemical structure. Thermal behaviour was investigated using differential scanning calorimetry and thermogravimetry coupled with FTIR gas analysis (TG-FTIR). The flammability performance of the epoxy blends was evaluated using pyrolysis combustion flow calorimetry, which allowed parameters such as heat release rate, total heat release, and heat release capacity to be determined. The incorporation of melamine phosphate-based flame retardants was found to significantly reduce the flammability of epoxy blends, leading to substantial decreases in heat release rate, total heat release, and heat release capacity. The most pronounced effect was observed in systems containing higher concentrations of MP and in cooperative ZnB-MP formulations.

## 1. Introduction

Epoxy resins are one of the most important groups of polymeric materials in modern materials engineering. Owing to their exceptional physicochemical properties, including high mechanical strength, excellent adhesion to various substrates, and high chemical resistance, they are employed in numerous industries [1,2,3,4,5,6]. Despite their numerous advantages, a significant limitation to their use remains their relatively high flammability, which poses a risk in environments with an increased fire hazard [7,8].

In response to the growing demand for fire safety and environmental protection, there is a growing focus on modifying epoxy resins by incorporating flame-retardant additives, particularly those derived from renewable materials [9,10,11,12]. Flame-retardant systems containing phosphorus and nitrogen are of particular interest as they exhibit a cooperative effect during combustion, enabling the reduced use of traditional halogenated flame retardants, which are considered a source of toxic gases and smoke during a fire [13,14,15]. The stable carbon layer formed by these compounds acts as a protective barrier to limit feedback from the combustion zone [16,17,18]. Currently, there is increasing emphasis on developing sustainable, halogen-free flame-retardant systems that incorporate biomass-based additives to reduce the rate of polymer pyrolysis and limit the formation of volatile fuel fragments. Due to their availability, chemical properties, and lower environmental impact, these additives represent a promising alternative to conventional solutions used in polymeric materials [19,20]. Recent studies have demonstrated the increasing potential of halogen-free, bio-based flame retardants for use with epoxy-based materials. For example, a bio-based flame retardant has been developed for epoxy resin/cyanate ester copolymers. This improves fire behaviour and reduces heat release while preserving favourable dielectric and thermal properties [21]. Other studies have also emphasised the growing importance of sustainable nanohybrid engineering methods in enhancing the performance of polymeric materials by encouraging cooperative interactions between fillers and interface design [22].

Among flame-retardant additives, melamine compounds and their phosphorus derivatives are commonly used in multi-component flame-retardant systems for polymeric materials [23,24,25]. Their effectiveness stems from the presence of nitrogen and phosphorus in their chemical structure, enabling them to act in both the gaseous and condensed phases simultaneously. Furthermore, hybrid flame-retardant systems combining melamine phosphates and zinc borates can improve flame-retardant properties through a cooperative effect [26].

Recent studies have increasingly emphasised the importance of multifunctional and cooperative flame-retardant systems that can improve both fire safety and the mechanical performance of polymeric materials simultaneously. A CuAl-layered double hydroxide/black phosphorus nanohybrid was developed for polyurea, in which black phosphorus acted as a gas-phase radical scavenger, while the CuAl-LDH promoted catalytic charring in the condensed phase. Consequently, the resulting material exhibited significantly reduced heat release and smoke production, as well as enhanced mechanical performance, thereby demonstrating the effectiveness of cooperative hybrid strategies in flame-retardant polymer design [27]. Similarly, the development of a halloysite-based nanohybrid featuring an engineered interface containing amino trimethylene phosphonic acid and silver nanocubes demonstrated its potential for application in polyurea composites. This hybrid system was found to significantly improve flame retardancy and smoke suppression, while also enhancing mechanical properties and providing antibacterial activity. This highlights the growing interest in multifunctional flame-retardant materials [28].

In epoxy systems, the effect of a phosphorus-containing melamine cyanurate derivative on the flammability and mechanical properties of an epoxy resin has been investigated. The presence of this compound significantly improves the material’s fire resistance, limiting the rate of heat and smoke release while simultaneously increasing its mechanical strength and glass transition temperature [29].

A novel triazolyl-based cyclomatrix polyphosphazene that self-assembles with melamine cyanurate has been synthesized. The presence of this compound significantly improves the fire resistance of epoxy resin, including reducing the rate of heat and smoke release, while also enhancing its mechanical properties and thermal stability [30].

The combined effect of microencapsulated red phosphorus coated with melamine borate and zinc borate as flame retardants for polyethylene has been explored. Using this system significantly improves the material’s fire resistance by forming a stable char layer and releasing non-flammable gases, thereby limiting the intensity of gas-phase combustion [31]. 

The cooperative effects of aluminium hydroxide, melamine, and zinc borate as flame retardants for epoxy resin have been investigated. A system containing aluminium hydroxide and melamine offers good fire resistance and improves the material’s thermal stability. This was evidenced by a reduction in the heat release rate and an increase in the limiting oxygen index [32].

However, despite substantial progress in multifunctional flame-retardant systems, developing efficient, halogen-free flame retardants for epoxy resins based on melamine phosphate derivatives remains challenging. In particular, the cooperative interactions between melamine phosphate compounds and inorganic flame-retardant additives, such as zinc borate, in epoxy systems are not well understood, especially with regard to their influence on thermal stability and fire behaviour while maintaining material performance.

An attempt to modify epoxy blends cross-linked with amine-derived curing agents by incorporating flame-retardant additives of melamine phosphate obtained through reactions between melamine and selected phosphorus derivatives has been carried out. A hybrid flame-retardant system combining melamine phosphate and hydrated zinc borate was also developed in order to evaluate its potential to enhance the thermal stability of, and improve the flame-retardant performance of, Epidian-based epoxy materials.

The resulting compounds were then incorporated into epoxy systems. The effect of their presence on the materials’ thermal properties, flammability and hardness was investigated. This analysis included differential scanning calorimetry (DSC), thermogravimetry (TG) and flammability tests to provide a comprehensive evaluation of the materials’ thermal stability and fire behaviour at elevated temperatures, as well as Shore hardness measurements.

## 2. Materials and Methods

### 2.1. Chemicals

Epidian® 601 (Epx601) epoxy resin, manufactured by CIECH Sarzyna S.A. (Nowa Sarzyna, Poland), is a clear, viscous liquid with a light yellow colour. Its epoxy number is 0.50–0.55 mol/100 g, and its viscosity at 25 °C is 700–1100 mPa · s. CIECH Sarzyna S.A. is also a manufacturer of the IDA curing agent, a transparent liquid with low viscosity (at 25 °C: 150–300 mPa · s) and an amine value of 200–350 mg KOH/g. IDA was obtained in the reaction of 3-(aminomethyl)-3,5,5-trimethylcyclohexan-1-amine (in excess) with epoxide based on 4,4′-(propane-2,2-diyl)diphenol (bisphenol A) and 1-chloro-2,3-epoxypropane. Roflam P, i.e., tris(1-chloropropan-2-yl) phosphate (TCPP) acting as a plasticizer, was purchased from PCC Rokita S.A. (Brzeg Dolny, Poland). Phosphoric acid, dibutyl hydrogen phosphate, bis(2-ethylhexyl) hydrogen phosphate, and melamine were obtained from Sigma-Aldrich, Darmstadt, Germany, and zinc borate hydrate (ZnB) was obtained from P.P.H. „STANDARD” Sp. z o. o., Lublin, Poland. 

**Figure 1:**
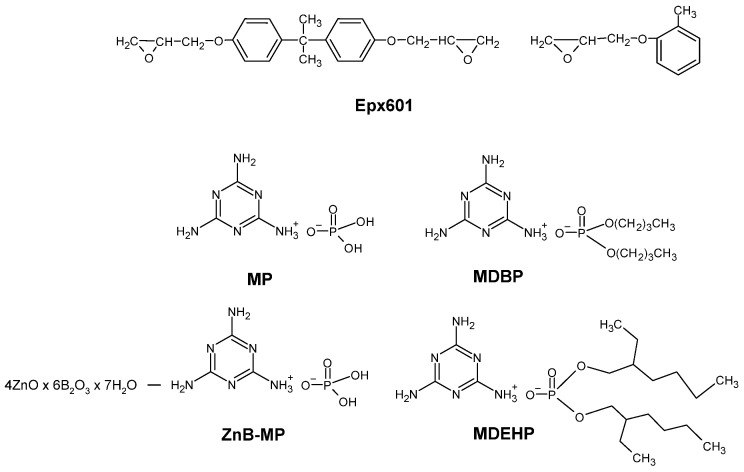
Chemical structures of the epoxy resin Epx601 and flame retardants used.

Chemical structures of the epoxy resin Epx601 and the flame retardants, i.e., melamine phosphate (MP), melamine dibutyl phosphate (MDBP), melamine di(2-ethylhexyl)phosphate (MDEHP), and a mixture of hydrated zinc borate–melamine phosphate with a 1:1 mass ratio (ZnB-MP), are given in Figure 1.

### 2.2. Flame-Retardant Preparation

#### 2.2.1. Preparation of MP, MDBP, and MDEHP Phosphates

Melamine phosphates were prepared by treating 12.60 g (0.1 mol) of melamine with 0.1 mol of the appropriate acid: (9.80 g of phosphoric acid), 21.02 g of dibutyl hydrogen phosphate, or 32.24 g of bis(2-ethylhexyl) hydrogen phosphate in 100 cm^3^ of water. After mixing the reagents for 20 min under stirring at 600 rpm, the mixture was heated at 95 °C for 2 h. The solution was then cooled to 10 °C for 24 h, and the resulting precipitate was filtered under reduced pressure and dried at 100 °C until constant weight was achieved. The yield of the reaction was 95–96%.

#### 2.2.2. Preparation of the ZnB-MP 

A mixture of 10.00 g of melamine phosphate, 10.00 g of hydrated zinc borate, and 10 cm^3^ of water was placed in a mechanical grinder operating at 1000 rpm and mixed for 20 min, followed by transfer to a mortar. The mixture was ground for 15 min at 25 °C and subsequently dried at room temperature (48 h) and then at 100 °C until a constant mass was obtained. The yield of the reaction was 98.5–99%.

The reaction yields and flame-retardant properties are presented in Table 1.

### 2.3. Blend Preparation

To obtain the blends tested in this study, epoxy resin, plasticizer, and flame retardant were added to a glass vessel in the amounts given in Table 2. 

**Figure 2:**
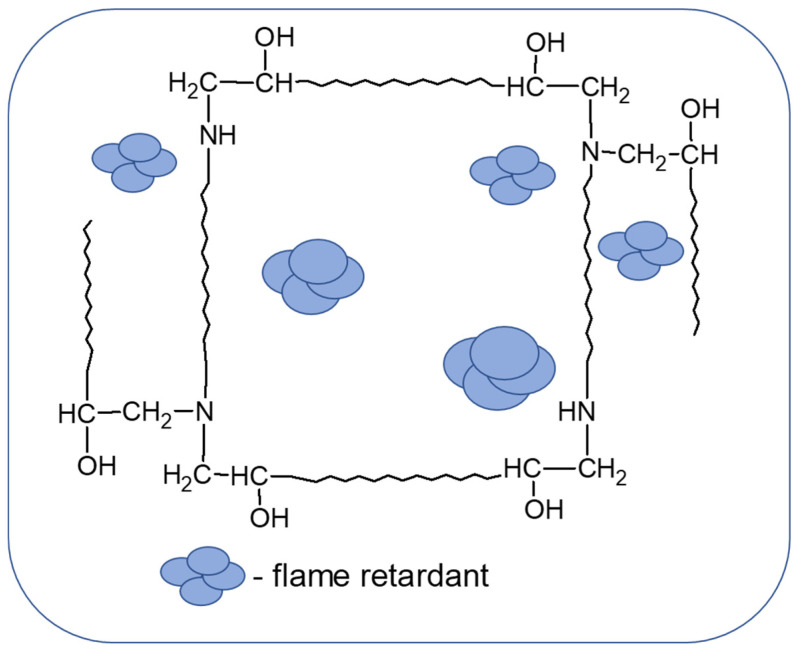
Schematic illustration of an epoxy/flame-retardant composition.

The resin was placed in a beaker, and an appropriate amount of flame retardant was added. The mixture was then thoroughly mixed with a stirring rod and placed in a heating chamber at 50 °C for 0.5 h to deaerate and homogenise the system. At room temperature, the IDA hardener was then added. The vessel contents were then gently stirred and returned to the heating chamber (50 °C) for approximately 10 min. The prepared composition was then poured onto 100 mm and 80 mm glass plates equipped with Teflon spacers and allowed to cure at 25 °C for 24 h, followed by 4 h at 80 °C in a heating chamber. In Figure 2, a schematic diagram of an epoxy composition with a flame retardant is presented. The yield of the reaction was 98.5–99%.

### 2.4. Measurement Methods

#### 2.4.1. Fourier Transform Infrared Spectroscopy (FTIR)

A Bruker Tensor 27 FTIR spectrometer (Hanau, Germany) with an attenuated total (internal) reflectance (ATR) module was applied to acquire the FTIR spectra. The spectrometer was equipped with a PIKE measuring cell, which features a crystalline diamond embedded in zinc selenide. Thus, the spectra were recorded within the range of 4000 cm^−1^ to 600 cm^−1^. The spectral resolution was 4 cm^−1^, and 32 scans were performed for each spectrum. The analyses were followed by measurements of the background spectrum.

#### 2.4.2. Thermal Properties

##### Differential Scanning Calorimetry (DSC)

DSC research was performed with a Netzsch 204 F1 Phoenix calorimeter (Netzsch, Günzbung, Germany). The measurements were done in aluminium crucibles with a pierced lid (mass of 40 ± 1 mg). As references, empty aluminium pans were used. Dry argon gas with a flow rate of 30 cm^3^/min was purged through the sample cell. Cooling was accomplished with liquid nitrogen. Samples of 11 ± 0.2 mg were first cooled to −100 °C, held at this temperature for 3 min, then heated to 200 °C, cooled once more to −100 °C, and reheated to 200 °C. The heating/cooling rate of 10 °C/min was used. The reported transitions are from the first and second heating cycles. The inflection point on the curves of the heat capacity changes has been chosen to represent the *T*_g_s for the samples.

##### Thermogravimetry (TG)

A Netzsch STA 449 F1 Jupiter instrument (Netzsch, Selb, Germany) was used to perform TG tests. Measurements were done in a flow of 20 cm^3^/min synthetic air (80% nitrogen, 20% oxygen). The samples were heated from 30 °C to 700 °C or 800 °C at a rate of 10 °C/min. The samples of 11±0.1 mg were placed in the open crucibles (made from Al_2_O_3_). As a reference, an empty Al_2_O_3_ crucible was used. The components of the gas generated during heating of the samples were analysed using a Bruker Tensor 27 FTIR spectrometer (Bruker, Hanau, Germany) connected online to a Netzsch STA instrument via a two-millimeter-diameter Teflon transfer line preheated to 200 °C. FTIR spectra were collected over the 600–4000 cm^−1^ spectral range, with 16 scans per spectrum and a resolution of 4 cm^−1^.

#### 2.4.3. Flammability Test with the Use of Pyrolysis Combustion Flow Calorimetry (PCFC) 

The flammability of the samples was examined using a PCFC microcalorimeter supplied by Fire Testing Technology Ltd. of East Grinstead, UK. The test was carried out in accordance with ASTM D 7309 [33]. The pyrolyzer temperature was 550 °C with a heating rate of 1 °C/s, and the combustion chamber temperature was 900 °C. The test was performed under nitrogen/oxygen conditions (80/20 cm^3^/min). 

#### 2.4.4. Hardness

The hardness of the samples was measured by the Shore D method on a Zwick 7206/H04 analogue hardness testing apparatus (ZwickRoell, Ulm, Germany). Measurements were taken after 15 s at 23 °C according to the ISO 868 standard [34]; taking five measurement points and calculating the mathematic average of them, which was regarded as the hardness value. 

## 3. Results and Discussion

Sixteen systems of blends were obtained, differing in type (MP, MDBP, MDEHP, and ZnB-MP) and content (8 mas%, 10 mas%, 15 mas%, and 25 mas%) of flame retardant. A sample without an added flame retardant was also prepared and used as a reference sample.

### 3.1. Spectroscopic Characteristics

The chemical structure of all prepared blends, the reference sample, and the main chemicals used in their synthesis were confirmed by using the ATR-FTIR method. Figure S1 presents the spectra found for the pure epoxide resin and all flame retardants.

The Epx601 spectrum (Figure S1) displays absorption bands typical of a hydroxyl group (at 3500 cm^−1^, O-H stretching vibrations); an aromatic ring (at 1606 cm^−1^, 1582 cm^−1^, and 1496 cm^−1^, C-C stretching vibrations; at 828 cm^−1^, C-H bending vibrations of the *p*-substituted benzene ring); methylene and methyl groups (at 2966 cm^−1^, asym. C-H stretching vibrations of the methyl group; at 2927 cm^−1^, asym. stretching vibrations C-H of the methylene group; at 2873 cm^−1^, sym. C-H stretching vibrations of both groups), and an epoxy ring (at 915 cm^−1^, C-H bending vibrations). The absorption bands visible at 3057 cm^−1^ and 3039 cm^−1^ are the result of the C-H stretching vibrations of the benzene and epoxy rings. Moreover, the band at 1455 cm^−1^ can originate from both the C-C stretching vibrations of the benzene ring and the C-H bending vibrations of alkylene groups. On the other hand, the band at 1240 cm^−1^ comes from the C-O stretching vibrations of an epoxide ring and the C_ar_-O-C_al_ stretching vibrations (from the bisphenol A unit). 

As regards the spectra recorded for the flame retardants (Figure S1), they all confirm the presence of a melamine unit and a phosphate group. The spectrum received for the MP sample shows absorption bands at 3369 cm^−1^ and 3120 cm^−1^, originating from the N-H stretching vibrations, whereas those at 1665 cm^−1^ and 1655 cm^−1^ come from the N-H bending vibrations. The existence of a triazine ring is indicated by bands located at 1560 cm^−1^, 1511 cm^−1^, 1478 cm^−1^ (the C-N stretching vibrations) and 777 cm^−1^ (the C-N bending vibrations). In turn, the bands at 1325 cm^−1^ (the P=O stretching vibrations) and 1003 cm^−1^ (the P-OH stretching vibrations) are evidence of the presence of a phosphate group. Moreover, it should be noted that the bands at 3369 cm^−1^ and 3120 cm^−1^ are also associated with the stretching vibrations of the O-H hydroxyl group [35,36,37]. In the MDBP and MDEHP spectra, additional distinct bands appeared, resulting from the C-H stretching vibrations of the methyl and methylene groups (at ~2960–2857 cm^−1^) and from the P-O stretching vibrations of P-O-C_al_ (at ~1012 cm^−1^). There are no bands connected with the presence of a hydroxyl group. In the ZnB-MP spectrum, bands visible for the MP sample are observed, characteristic of the amine group, phosphonate group, and triazine ring, with slight differences in wavenumber values. Nevertheless, absorption bands seen above 3000 cm^−1^ may also originate from the stretching vibrations of water contained in zinc borate. Also, the absorption bands at 1077 cm^−1^ and 681 cm^−1^, attributed to the B-O stretching and bending vibrations, respectively, are observed [38,39]. The Zn-O vibration band, which is typically observed at ~450 cm^−1^, exceeds the measuring range of the spectrometer used. 

Based on the above analysis, it can be concluded that the main absorption bands of flame retardants largely overlap with those of pure epoxy resin.

**Figure 3:**
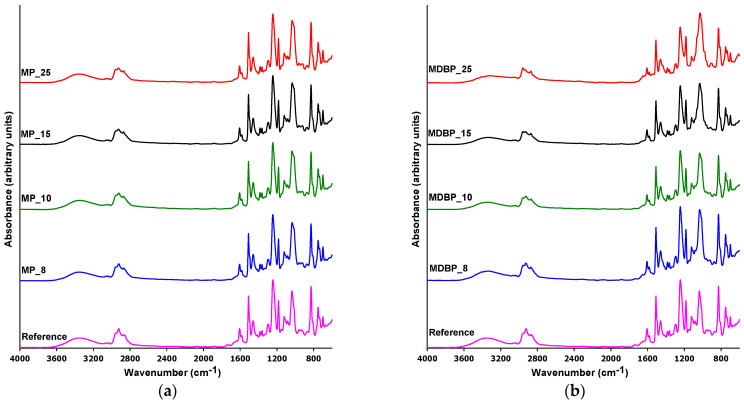

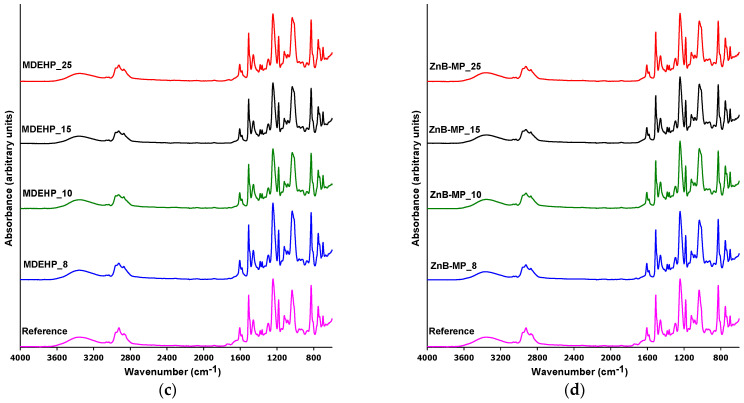
ATR-FTIR spectra of the blends prepared from MP (**a**), MDBP (**b**), MDEHP (**c**) and ZnB-MP (**d**), as well as the reference sample.

The spectra obtained for the reference sample and blends containing flame retardants are very similar (see Figure 3a–d). Thus, it can be said that the addition of a flame retardant to the polymer matrix does not significantly affect the ATR-FTIR spectra of the prepared blends. Cured epoxy resin is the basic component of the tested materials. The spectrum of the reference sample exhibits a wide absorption band at 3352 cm^−1^ coming from the O-H and N-H stretching vibrations. The presence of hydroxyl groups is also indicated by the C-OH stretching vibration band at 1150 cm^−1^, and the occurrence of amine groups is also evidenced by the N-H bending vibration band at 1509 cm^−1^. In addition, small bands are visible, which can be attributed to the C-H stretching vibrations (at 3140 and 3061 cm^−1^) and the C-C stretching vibrations (at 1606 cm^−1^) of benzene rings. The *p*-substituted benzene ring indicates a strong band at 827 cm^−1^, originating from the C-H bending vibrations. The spectrum also shows absorption bands in the range of 2957–2870 cm^−1^, associated with the C-H stretching vibrations in the methyl and methylene groups. The reference sample spectrum further reveals a band at 1244 cm^−1^ corresponding to the C-O stretching vibrations. However, no clear band at ~915 cm^−1^, indicating the presence of epoxy rings, was observed.

### 3.2. DSC Analysis

**Figure 4:**
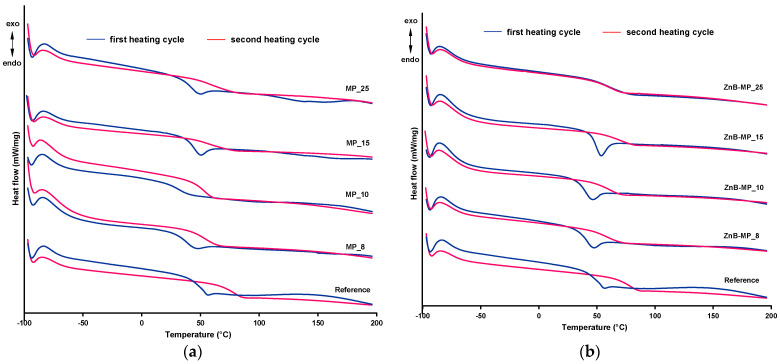
DSC thermograms of the blends of series MP (**a**) and ZnB-MP (**b**) and the reference sample.

**Figure 5:**
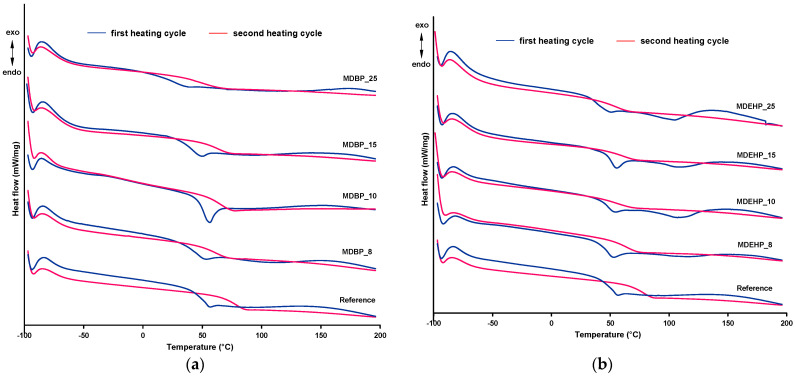
DSC thermograms of the blends of series MDBP (**a**) and MDEHP (**b**) and the reference sample.

DSC measurements were conducted for all prepared flame-retardant blends and the reference sample, being the polymer matrix in the blends. The received results are presented in Table 3 and Figure 4 and Figure 5.

The DSC thermogram obtained for the reference sample (Figure 4 and Figure 5) in the first heating cycle shows a distinct step corresponding to the glass transition (*T*_g_ = 54 °C) and a broad exothermic peak in the 100–200 °C range associated with the cross-linking process. This process was accompanied by a heat release (*ΔH*) of 11.26 J/g. In the second heating cycle, only a glass transition is observed, with *T*_g_ shifted to 78 °C. 

For all MP series blend samples (Figure 4a), a glassy transformation is seen, which, as with the reference sample, shifts toward higher temperatures during the second heating cycle, from 26–52 °C to 43–68 °C. An increase in *T*_g_ values in the second heating was also found for Epx601-based blends containing natural fillers in the form of minerals from the silicate cluster kaolinite and clinoptilolite [40]. In the case of samples with a high flame-retardant content (MP_15 and MP_25), broad and slight endotherms, in the range of approximately 100–200 °C, are additionally observed on the thermograms obtained during the first heating cycle, related to the melting of MP (*ΔH* = 4.76 J/g and 7.11 J/g, respectively), which disappear in the second heating. No exothermic effects related to the cross-linking of blends are noticeable.

A lack of exotherms is also found in the DSC thermograms recorded for the ZnB-MP series blends (Figure 4b). The curves reveal only glass transitions. The *T*_g_ values in the first heating cycle range from 38 °C to 49 °C, and in the second one from 57 °C to 62 °C.

The situation is different for the other two series of blends (Figure 5a,b). Neither the addition of MDBP nor MDEHP inhibited the cross-linking process in these materials. In the case of MDBP-based blends, the heat of this process ranges from 4.74 J/g to 11.90 J/g and does not depend directly on the amount of flame retardant used. However, the lowest curing heat was detected for the MDBP_25 sample, which contains the highest amount of flame retardant. In turn, for blends prepared from MDEHP, the highest curing heat value (18.99 J/g) was observed for the sample containing the highest amount of flame retardant (MDEHP_25), and the lowest one for the MDEHP_8 sample (4.21 J/g). The DSC curves registered for materials derived from MDEHP also show endotherms (in the range of about 75−135 °C) that precede the exotherms. These endothermic transitions can be attributed to the melting of the MDEHP flame retardant and are characterized by *Δ*H values from 3.78 J/g to 9.23 J/g. Slightly lower *T*_g_ values were determined for the MDEHP-based blends than for the MDBP-based ones (38−45 °C vs. 39−49 °C during the first heating and 52−73 °C vs. 58−74 °C during the second heating). When analysing the effect of the addition of a flame retardant on the *T*_g_ value, it is difficult to clearly determine the relationships in all series of obtained blends. 

The *T*_g_ values of these new Epidian 601-based blends are generally lower than those determined for similar blends made from Epidian 601 resin cross-linked with IDA, PAC, or triethylenetetramine curing agents, which contained phosphorus-based fire retardants (triphenyl phosphate or Fire Retardant 421 from West System) [41]. 

The post-cross-linking effect observed in MDBP and MDEHP systems may be due to the presence of free phosphate acid groups. These groups can interact with amine curing agents, reducing their reactivity towards epoxy groups. Consequently, incomplete curing occurs during the initial cross-linking stage, resulting in residual epoxy groups that undergo further reaction when heated. In contrast, the ZnB-MP system does not exhibit this behaviour, likely because the phosphate species associated with the inorganic zinc borate phase have reduced mobility and lower chemical activity. The reduced reactivity of amine curing agents in epoxy systems, coupled with the significant impact of hydrogen-bonding interactions, is a well-documented phenomenon in the literature. These bonds can significantly impact the curing kinetics and advancement of the epoxy–amine reaction, resulting in changes to network formation and material properties. In particular, Mora et al. [42] demonstrated that hydrogen bonding plays a crucial role in modulating amine reactivity, altering the reaction pathway in epoxy–amine systems by influencing molecular mobility and the accessibility of reactive functional groups.

### 3.3. TG-FTIR Analysis

**Figure 6:**
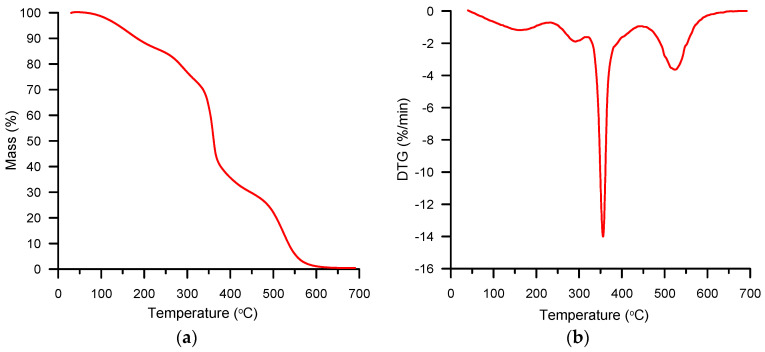
TG (**a**) and DTG (**b**) curves obtained for the reference sample.

**Figure 7:**
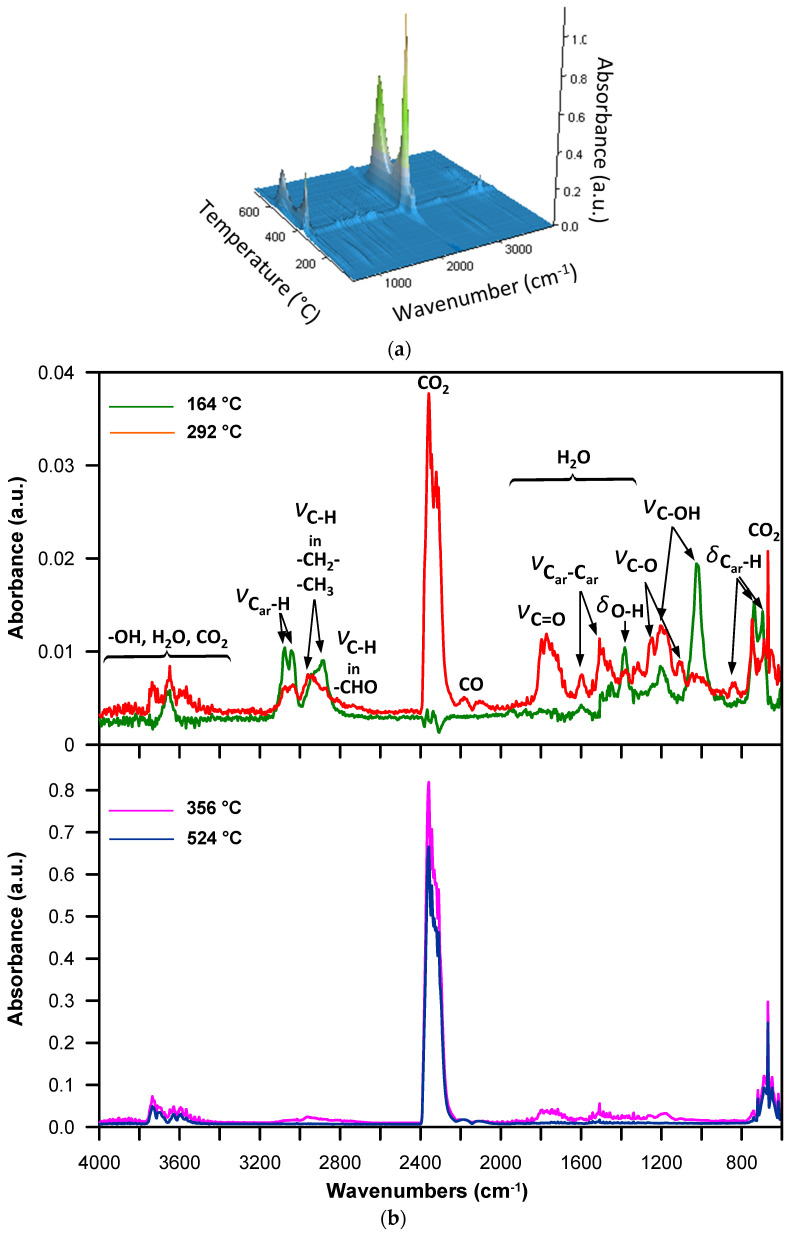
FTIR spectra of gaseous products evolved during thermal decomposition of the reference sample: (**a**) 3D and (**b**) extracted at the maxima of decomposition.

**Figure 8:**
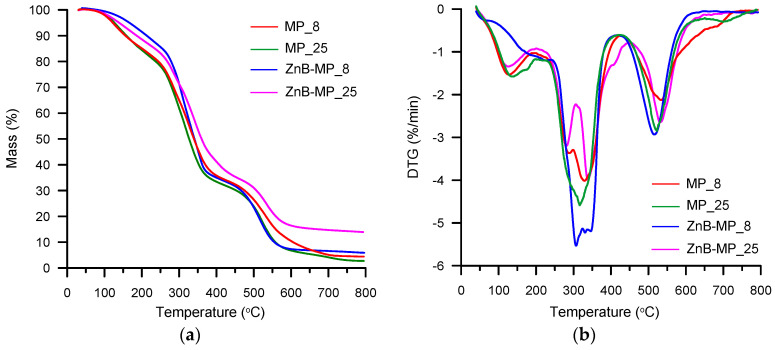
TG (**a**) and DTG (**b**) curves of the blends of series MP and ZnB-MP.

**Figure 9:**
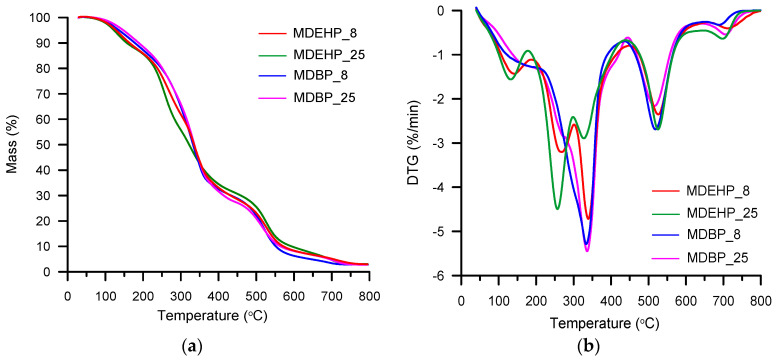
TG (**a**) and DTG (**b**) curves of the blends of series MDBP and MDEHP.

**Figure 10:**
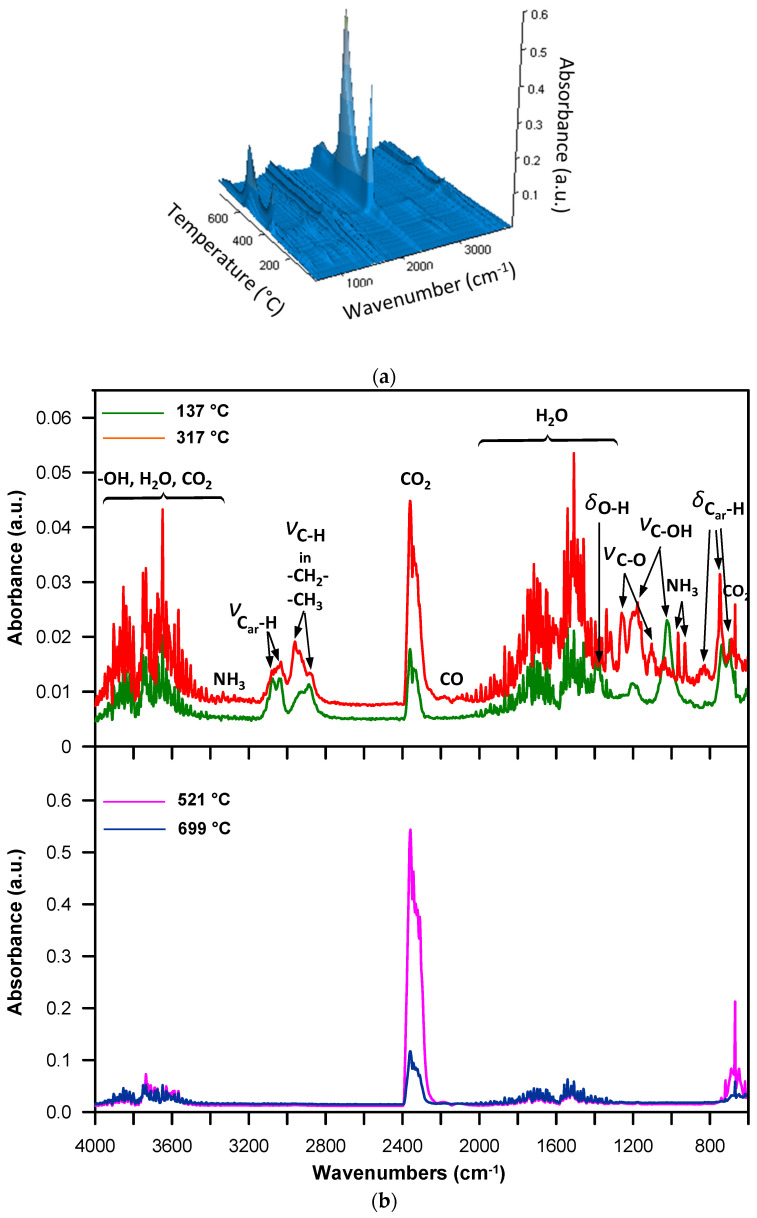
FTIR spectra of gaseous products evolved during thermal decomposition of the MP_25 sample: (**a**) 3D and (**b**) extracted at the maxima of decomposition.

**Figure 11:**
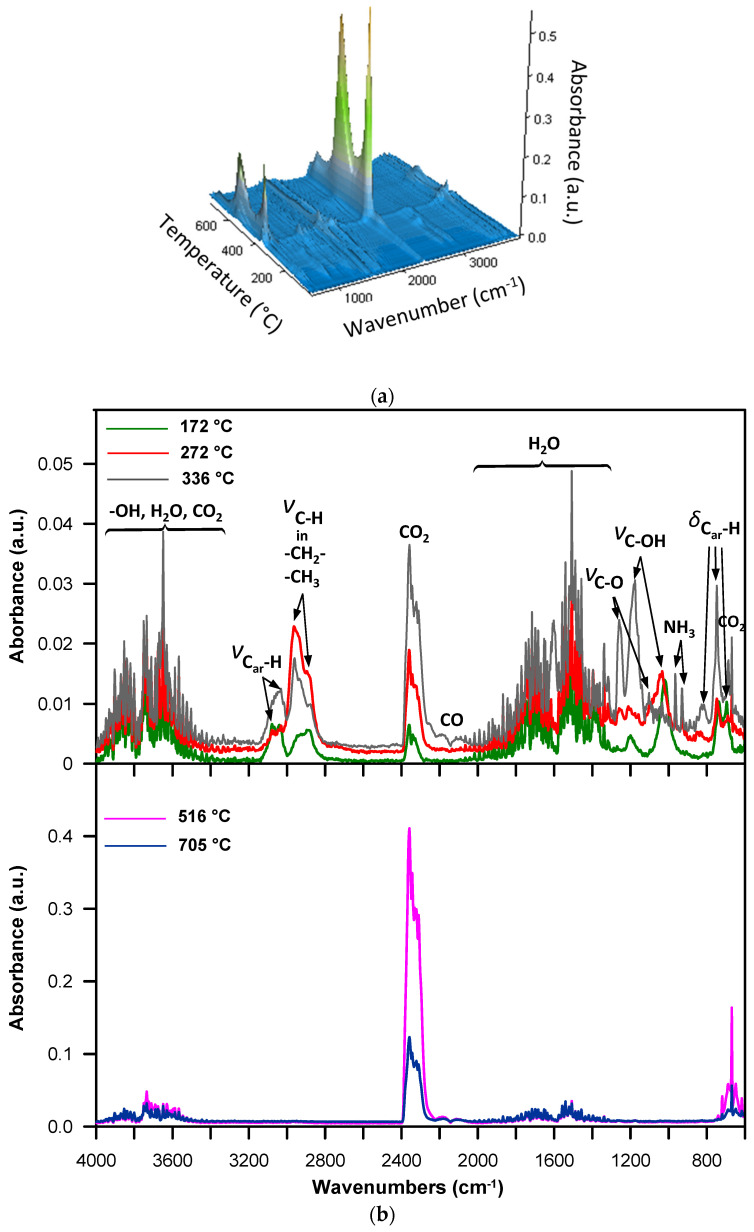
FTIR spectra of gaseous products evolved during thermal decomposition of the MDBP_25 sample: (**a**) 3D and (**b**) extracted at the maxima of decomposition.

**Figure 12:**
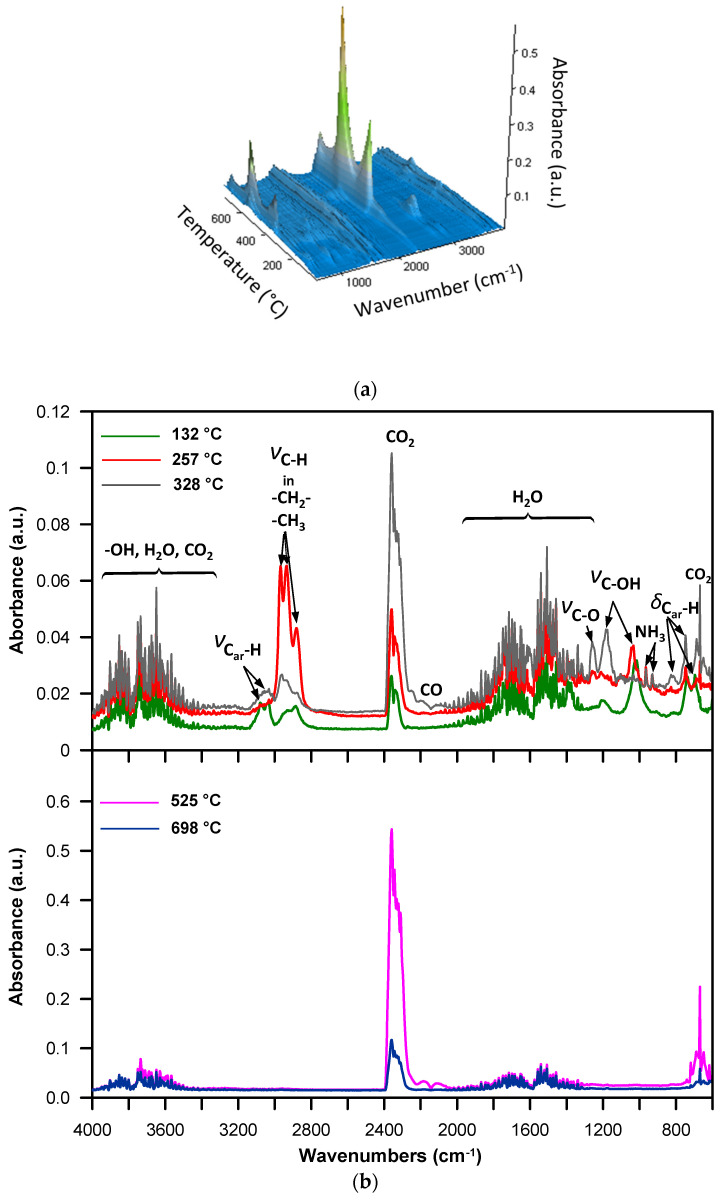
FTIR spectra of gaseous products evolved during thermal decomposition of the MDEHP_25 sample: (**a**) 3D and (**b**) extracted at the maxima of decomposition.

**Figure 13:**
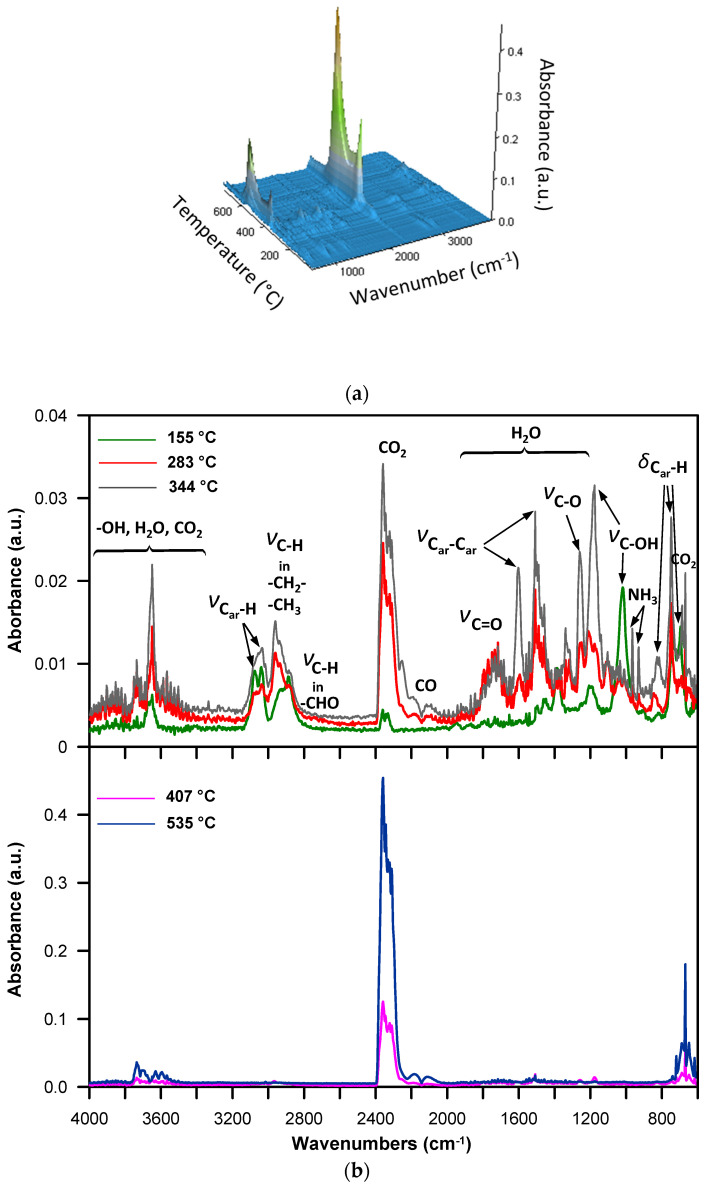
FTIR spectra of gaseous products evolved during thermal decomposition of the ZnB-MP_25 sample: (**a**) 3D and (**b**) extracted at the maxima of decomposition.

To demonstrate the effect of the lowest and highest amounts of flame retardant used on the thermal stability of the prepared mixtures, TG tests were conducted on samples containing 8% by mass and 25% by mass of flame retardant, as well as on a reference sample. The courses of the recorded TG and differential TG (DTG) curves are presented in Figure 6, Figure 8 and Figure 9. Based on the TG curves, the temperatures at which there are mass losses of 5% (*T*_5_), 10% (*T*_10_), and 50% (*T*_50_) were read. In turn, the temperature at which the maximum rate of mass loss occurs (*T*_max_) for particular decomposition stages was determined on the basis of DTG curves. The approximate mass loss corresponding to a given stage of decomposition and the mass of the sample remaining after the decomposition process were also determined. These indicators are listed in Table 4. Furthermore, the gaseous products released during thermal decomposition were analysed using an FTIR spectrometer for the reference sample and for blends with a 25 mass % flame retardant content. Figure 7, Figure 10, Figure 11, Figure 12 and Figure 13 show the 3D spectra recorded within the entire temperature range and those extracted at *T*_max_ for each decomposition stage.

The decomposition of all tested samples in a synthetic air atmosphere is a multi-stage process that proceeds in four or five overlapping stages.

In the case of the reference sample (Figure 6), the first stage with *T*_max_ = 164 °C is associated with a 14% loss in mass. In this stage, the compounds released are primarily aliphatic, aromatic, and those containing hydroxyl groups (see the FTIR spectrum collected at 164 °C (green line) presented in Figure 7b). The formation of aliphatic products is evidenced by the bands at 2935–2883 cm^−1^, corresponding to the stretching vibrations of C-H bonds in methyl and methylene groups, while the emission of monosubstituted aromatic products is confirmed by the bands at 3076–3044 cm^−1^ and 737–696 cm^−1^, associated with the C-H stretching and bending vibrations, respectively, as well as at 1598 cm^−1^, connected to the C-C stretching vibrations. The presence of products with hydroxyl groups is manifested by the bands at 3660 cm^−1^ and 1382 cm^−1^, originating from the O-H vibrations, specifically stretching and bending, as well as the bands at 1206 cm^−1^ and 1025 cm^−1^, referring to the C-OH stretching vibrations in alcohols and phenols, respectively. In addition, small amounts of carbon dioxide (bands at 2358–2310 cm^−1^, connected to the asymmetric stretching vibrations) and water (bands at ~4000–3500 cm^−1^ and ~1800–1300 cm^−1^, associated with the stretching and bending vibrations, respectively) are emitted. In the second stage of decomposition (*T*_max_ = 292 °C, a 12% loss in mass), significantly smaller amounts of alcohols and phenols are evolved, but much larger amounts of carbon dioxide are produced. Furthermore, there are emissions of carbon monoxide (bands at 2182 cm^−1^ and 2106 cm^−1^) and organic carbonyl compounds (bands at 1796–1700 cm^−1^—the C=O stretching vibrations), including aldehydes (bands at ~2820 cm^−1^ and 2740 cm^−1^—the C-H stretching vibrations) and esters (bands at 1245 cm^−1^ and 1000 cm^−1^—the C-O stretching vibrations). The spectrum from this stage (red line in Figure 7b) also shows bands associated with the evolution of p-disubstituted benzenes (bands at 847–833 cm^−1^—the C-H bending vibrations). The creation of aromatic compounds is also indicated by the band at 1507 cm^−1^, related to the C-C stretching vibrations. In turn, during the third stage (*T*_max_ = 356 °C), the main decomposition of the sample occurs, accompanied by the greatest loss of mass, amounting to 43%. Among the detected products (see the FTIR spectrum recorded at 356 °C (pink line) shown in Figure 7b) are mainly carbon dioxide, carbon monoxide, organic carbonyl compounds, and aromatics. The fourth stage of the reference sample’s decomposition (*T*_max_ = 524 °C, a 29% loss in mass) involves only oxidative reactions of the char residue formed during the previous decomposition stages. This is proven by the release of gaseous products such as carbon dioxide, carbon monoxide, and water vapor, as can be visible in the FTIR spectrum recorded at 524 °C (blue line in Figure 7b). It should be noted that the sample decomposed almost completely (residual mass at 700 °C = 1.6%). Considering *T*_5_ as a main thermostability criterion, it can be said that the reference sample is stable up to 141 °C. In turn, 10% and 50% mass losses were recorded at 184 °C and 362 °C, respectively.

As can be seen from the data presented in Table 4, the blends based on MP, MDBP, and MDEHP exhibit lower resistance to heating than the reference sample. The *T*_5_, *T*_10_, and *T*_50_ values are lower, at 122–140 °C, 156–182 °C, and 322–340 °C, respectively. A similar situation occurred with epoxy resins based on bisphenol A that were modified with MP. They decomposed at relatively lower temperatures than those without MP. The determined *T*_5_ and *T*_10_ values were lower by approximately 13–78 °C and 13–65 °C, respectively [43]. A decrease in thermal stability following the addition of a 9,10-dihydro-9-oxa-10-phosphaphenanthrene-10-oxide derivative as a flame retardant was also observed in bisphenol A-based epoxide resins cured with 4,4′-diaminodiphenylmethane [44].

In contrast, the use of ZnB-MP as a flame retardant improved the initial thermal stability of the newly prepared blends compared to the reference sample, particularly in the case of the ZnB-MP_8 sample. The *T*_5_ values are 167 °C for ZnB-MP_8 and 143 °C for ZnB-MP_25, and the *T*_10_ values are 214 °C and 188 °C, respectively. Only the *T*_50_ values were reduced to 341 °C and 360 °C. The incorporation of a flame retardant into the polymer matrix resulted in an increase in the post-decomposition residue to 2.6–4.0% for the blends prepared from MP, MDBP, and MDEHP, and to 4.7% and 12.2% for those derived from ZnB-MP.

In the case of MDBP- and MDEHP-series blends, the char yield increases slightly as the phosphorus content increases. In the case of MP-series blends, no such relationship is observed. 

Among all of the investigated samples, ZnB-MP_25 exhibited the highest residual mass (12.2 mas%). This behaviour can be attributed to the presence of zinc borate, which forms thermally stable inorganic degradation products during decomposition, and the phosphorus-containing flame retardant, which promotes char formation. The combined action of these components results in an increased amount of residue remaining after thermal degradation.

The MP_25 blend decomposes in four stages that partially overlap (Figure 8), accompanied by volatile products whose FTIR spectra are shown in Figure 10. In the first stage, there is a 15% loss in mass. The FTIR spectrum recorded at *T*_max_ = 137 °C (green line in Figure 10b) reveals the same types of products that were identified for the reference sample. Specifically, bands characteristic of carbon dioxide and water, as well as aromatic, aliphatic, and hydroxyl compounds, are visible. In the second stage (*T*_max_ = 317 °C), during which the main decomposition of the blend occurs (52% mass loss), a significant increase in the intensity of bands originating from water and carbon dioxide is observed, as shown in Figure 10b (red line). Additionally, the spectrum shows bands typical of carbon monoxide, a *p*-disubstituted benzene ring (at 828 cm^−1^), and at 1103 cm^−1^ and 1255 cm^−1^, bands attributable to the C-O-C vibrations in esters, although due to the emission of large amounts of water, the C=O stretching vibration band is not visible. Carbon monoxide, esters, and p-disubstituted benzenes have previously been detected in the reference sample. New bands are bands indicating the emission of ammonia, a product of the decomposition of the MP fire retardant, at 965 cm^−1^ and 930 cm^−1^, which come from the N-H bending vibrations, and at 3300 cm^−1^, associated with the N-H stretching vibrations. A significant quantity of water may be due to the dehydration of MP into melamine pyrophosphates and melamine polyphosphates, which subsequently decompose to release ammonia and water, leading to the production of melamine pyrophosphates and melamine polyphosphates [36]. From the literature [45], it follows that the decomposition of the melamine ring at temperatures above 300 °C is accompanied by the absorption of heat from the surroundings as a result of nitrogen release. As a consequence, melamine phosphates are effective as flame retardants in polymeric materials that require processing at high temperatures or are intended for use at elevated temperatures. However, nitrogen is IR-inactive, so it cannot be detected in the recorded spectra. The last two stages (*T*_max_ = 521 °C and 699 °C), during which a total of 30% of the blend decomposes, involve the emission of large amounts of carbon dioxide (particularly in the third stage), carbon monoxide, and water—all of which are products of the oxidation reactions taking place.

The thermal decomposition of MDBP_25 proceeds in five partially superimposed stages (Figure 9 and Table 4) and begins slightly earlier than that of the reference sample. The *T*_5_ of this blend is 140 °C, which is only 1 °C lower than the *T*_5_ value recorded for the reference sample. The first stage, with a maximum at 172 °C, and a mass loss of approximately 12%, is associated with the evolution of the same kinds of compounds as in the case of the reference sample, i.e., those containing aromatic and aliphatic species, hydroxyl groups, as well as carbon dioxide and water (see the FTIR spectrum collected at 172 °C (green line) in Figure 11b). The second one, with the maximum at 272 °C, is visible as a shoulder. During this stage, the blend loses approximately 17% of its mass. The aforementioned products are emitted with significantly higher amounts of aliphatic compounds and carbon dioxide. Small amounts of ammonia and esters are also released. In the next stage, with a maximum at 336 °C, the same products are formed as in the second stage; the differences lie solely in the quantities. A comparison of the spectra obtained for MDBP_25, MP_25, ZnB-MP_25, and the reference sample clearly shows that more aliphatic compounds were formed during the decomposition of the MDBP_25 sample, which is due to the presence of dibutyl groups in the fire retardant used. The FIIR spectra from the third and fourth stages (*T*_max_ = 516 °C and 705 °C) are very similar in appearance to those of the MP_25 sample, indicating a similar decomposition profile.

Like the MDBP_25 sample, the MDEHP_25 blend decomposes in five overlapping stages (Figure 9). However, it is slightly less thermally stable than MDBP_25. The *T*_5_, *T*_10_, and *T*_50_ values are several degrees Celsius lower than those obtained for MDBP_25. As for the decomposition products released (Figure 12), the FTIR spectra obtained show a similar appearance. A clear difference is visible in the intensity of the bands associated with the stretching vibrations of aliphatic C-H groups. The bands observed in the MDEHP_25 sample spectrum have greater intensity, which is consistent with the structure of the fire retardant used. 

The decomposition of the ZnB-MP_25 blend takes place in one more stage than that of the reference sample (Figure 8). Five peaks are observed on the DTG curve, with maxima at 155 °C, 283 °C, 344 °C, 407 °C, and 535 °C. The FTIR spectra (green and red lines in Figure 13b) recorded during the first two stages, in which 30% of the blend decomposes, are essentially similar and indicate the emission of mostly the same products. In addition, ammonia was released. In the third stage, in which a 28% mass loss occurs, significantly more esters, aromatic and hydroxyl compounds, and ammonia are released than in the second one. No formation of new products is observed. In the fourth stage (*T*_max_ = 407 °C, a 9 % mass loss), which was not observed for the reference sample, aromatic and aliphatic compounds, esters, carbon dioxide, carbon monoxide, ammonia, and water continue to form. The last one (a 21% mass loss) is the emission of water, carbon monoxide, and carbon dioxide. The residue left at 800 °C is 12%. It should be noted that the ZnB-MP_25 blend exhibits better thermal stability than the reference sample, as evidenced by the measured values of *T*_5_ and *T*_10_.

### 3.4. Flammability Test

**Figure 14:**
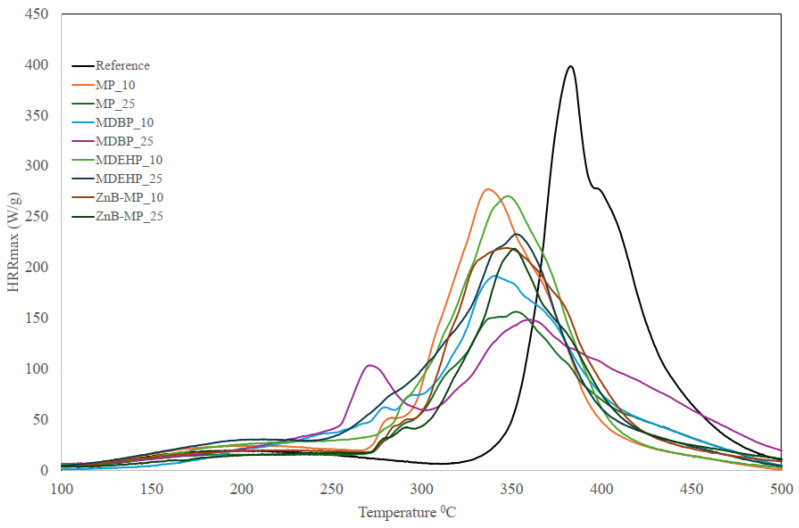
Flammability test of the studied samples.

A flammability test involving PCFC was done for selected blends, i.e., those containing 10 mas% and 25 mas% of a flame retardant, as well as for a reference sample. The data obtained are given in Table 5 and Figure 14.

The following parameters were recorded during the test: maximum heat release rate (HRR_MAX_) (W/g), time to HRR_MAX_ (THRR_MAX_) (s), total heat release (THR) (kJ/g), and heat release capacity (HRC) (J/gK). 

The results presented in Table 5 and Figure 14 demonstrate a clear reduction in the flammability of the investigated polymer blends, as evidenced by decreases in the HRR_MAX_, THR, and HRC values. 

The incorporation of MP into the epoxy resin matrix leads to a significant reduction in both HRR and HRC values. Notably, for blends containing MP, the decrease in these parameters is proportional to the MP content within the polymer matrix. The addition of 10 phr (parts per hundred resin) of MP results in a 29% reduction in HRR_MAX_, whereas increasing the MP content to 25 phr yields a reduction of up to 61%. HRR_MAX_ is a fundamental parameter for assessing the fire hazard of polymer blends. Higher HRR_MAX_ values correspond to more rapid thermo-oxidative decomposition of the material and, consequently, to more intense generation of volatile degradation products that sustain combustion. 

It should also be noted that the THRR_MAX_ parameter for all tested samples is lower than for the reference sample. This indicates that samples containing flame retardants are characterized by lower thermal stability, especially in the decomposition temperature range, compared to the reference sample. The obtained test results (microcalorimetry method) correlate well with the thermal stability results presented in Table 4 (*T*_50%_ parameter). 

HRC is similarly critical in evaluating fire hazard. It is defined as the maximum specific heat release rate obtained during pyrolysis—combustion testing divided by the heating rate, and it reflects the maximum heat release per unit mass under defined thermal conditions. A strong correlation between HRC and HRR_MAX_ is typically observed, making both parameters reliable indicators of flammability. The substantial reduction in HRR_MAX_, HRC, and THR (particularly for sample MP_25) can be attributed to the decomposition mechanism of melamine phosphate. Within the temperature range of blend degradation, MP decomposes to release nitrogen-containing gaseous species. Nitrogen-based products, including ammonia and nitrogen oxides, enter the flame zone, where they dilute flammable volatiles and reduce the local oxygen concentration. The phosphate (phosphorus at high oxidation level) undergoes facile decomposition in the degrading polymer matrix to form acidic species, which promote cationic cross-linking and char formation at the surface of the polymer.

The data in Table 5 further indicate that MDBP exhibits markedly higher efficiency than MP in reducing HRR_MAX_, THR, and HRC. This enhanced performance can be attributed to its molecular structure. In MP, the phosphorus atom is bonded to hydroxyl groups, whereas in MDBP, it is incorporated within an ester functionality, which serves as an effective carbon source. This promotes the formation and stabilization of a carbonaceous char layer at the material surface during decomposition. 

In the case of MDEHP-filled systems, the phosphorus atom is also bonded to an ester group; however, the more extended structure of the ester moiety leads to its fragmentation via homogeneous decomposition. The resulting volatile carbon-containing products do not significantly contribute to flammability reduction, as reflected in the HRR_MAX_, THR, and HRC values.

A pronounced decrease in fire hazard is observed for ZnB-MP systems. This effect arises primarily from cooperative interactions between zinc borate and melamine phosphate in the condensed phase. During thermal decomposition, MP facilitates the formation of an expanded, carbon-stabilized surface layer, while zinc borate enhances its structural integrity. The relatively less favourable results obtained for ZnB-MP compared to MP alone are likely a consequence of the measurement methodology. In the PCFC method, the sample undergoes controlled pyrolysis, and the evolved gases are subsequently combusted; the associated heat release is recorded as HRR_MAX_, THR, and HRC. This approach does not adequately capture condensed-phase mechanisms. Therefore, the method primarily reflects the quantity of combustible volatiles generated rather than the protective effects of char formation. It can thus be inferred that the barrier layer formed in ZnB-MP systems limits the release of flammable gases into the combustion zone. Under real fire conditions, such as those simulated by cone calorimetry, the effectiveness of the ZnB-MP system would likely be significantly greater.

### 3.5. Thermal Degradation Mechanism

**Figure 15:**
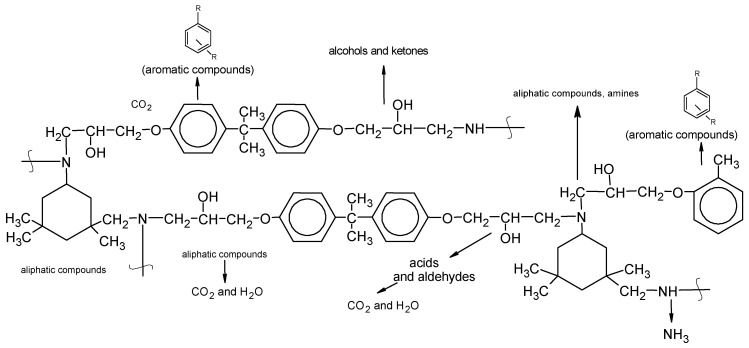
Schematic representation of the thermal fragmentation mechanism of the polymer under combustion conditions.

Figure 15 shows the proposed thermal degradation pathway of the epoxy–amine network during combustion. The initial stage of decomposition is expected to involve cleavage of the thermally less stable β-hydroxy ether and C-N bonds formed during the curing reaction between the epoxy resin and amine hardener. At the same time, aryl ether linkages may break down, creating phenolic compounds, substituted phenols, and fragments of bisphenol A. These aromatic intermediates can undergo further decomposition to form alkyl-substituted benzene derivatives and other aromatic hydrocarbons.

The degradation of the cycloaliphatic amine segments proceeds via C-N bond cleavage, deamination and ring-opening reactions. This leads to the formation of low-molecular-weight amines, ammonia, and aliphatic hydrocarbons. In parallel, fragments containing hydroxyl groups generated during chain scission may undergo dehydration and oxidation reactions to produce alcohols, ketones, aldehydes, and organic acids. Further oxidation of these intermediate species results in the evolution of gaseous products, including H_2_O, CO_2_, and trace amounts of CO. The proposed degradation pathway is consistent with the decomposition products identified during thermal analysis, as well as with the degradation mechanisms reported in the literature for epoxy–amine networks and phosphorus-containing flame-retardant systems [46,47,48,49].

### 3.6. Hardness Test

Shore D hardness measurements were performed on all prepared blends and the reference sample, and the resulting data are presented in Table 3. The data show that all blends have lower hardness than the reference sample (50.2–73.6 °Sh vs. 76.0 °Sh). In the MDBP and MDEHP series, the values decrease as the flame-retardant content increases, whereas in the other series, no such relationship is observed. The lowest hardness values (in the range of 50.2–69.1 °Sh) are exhibited by blends obtained from the flame retardant containing the highest number of aliphatic groups and, at the same time, the lowest phosphorus content (MDEHP). 

The newly received blends exhibit hardness values similar to or greater than those measured for blends made from Epidian 601-resin cured with IDA and triphenyl phosphate or Fire Retardant 421 from West System as a flame retardant [41].

## 4. Conclusions

The cross-linking reaction of Epidian 601 epoxy resin using IDA was used to prepare new blends containing melamine phosphate-based flame retardants, such as MP, MDBP, MDEHP, and a zinc borate-modified system (ZnB-MP). In addition to the type of flame retardants, the resulting blends also differed in their content (from 8 mas% to 25 mas%). Their chemical structure was verified using ATR-FTIR spectroscopy. 

From the DSC studied, it follows that the addition of a flame retardant to the polymer matrix caused a decrease in *T*_g_ in both the first (from 54 °C to 26–52 °C) and second (from 78 °C to 43–74 °C) heating cycles. Analysing the effect of incorporating flame retardants on the *T*_g_ value, it is hard to clearly identify any correlation in the series of blends produced. For two series, namely the MDBP and MDEHP series, the DSC curves exhibited exotherms associated with post-cross-linking in addition to the glass transition. In turn, endotherms related to flame-retardant melting were observed for some samples from the MP and MDEHP series.

Thermogravimetric analysis of epoxy blend materials conducted in a synthetic air atmosphere revealed a multi-step degradation process, as evidenced by the TG and DTG curves. These blends decomposed in four or five stages that overlapped. The main decomposition occurred at temperatures up to approximately 420 °C. Analysis of the volatile products using FTIR spectroscopy indicated that the decomposition of these materials was accompanied by the release of organic carbonyl compounds, such as aldehydes and esters, alcohols, phenols, aromatic and aliphatic substances, carbon dioxide, carbon monoxide, water, and ammonia. These volatile compounds were detected in all samples tested. Differences were observed mainly in the amounts of the respective compounds emitted. Above this temperature, the processes that took place were mainly oxidation reactions of the products formed in earlier stages of decomposition, as manifested by one or two peaks on the DTG curves. The products of these processes were carbon dioxide, carbon monoxide, and water. Residual masses after analysis were 3–4% for the MP-, MDBP- and MDEHP-based samples and 5–12% for the ZnB-MP-based ones.

The TG analysis also showed that the addition of ZnB-MP flame retardant improved the initial high-temperature resistance of the tested materials. The *T*_5_ and *T*_10_ values were higher than those of the reference sample, which consisted of a polymer matrix, and ranged from 143–167 °C and 188–214 °C, compared to 141 °C and 184 °C. In the case of the other blends based on MP, MDBP, and MDEHP, these temperature indicators decreased. However, the *T*_50_ values for each series of blends were lower than those of the reference sample and were in the range of 322–360 °C versus 362 °C. This corresponds well with the THRR_MAX_ parameter.

Moreover, it was found that the usage of all four selected melamine phosphate-based flame retardants significantly reduced the flammability of these epoxy blends, resulting in a significant decrease in the HRR_MAX_, THR, and HRC values. Nevertheless, the smallest reduction was observed for the MDEHP series. Considering the amount of flame retardant, it can be said that the fire hazard was diminished to a greater extent in samples containing 25 mas%. 

The hardness test revealed that adding a flame retardant to the epoxy resin reduces the hardness of the resulting blends. The greatest decrease in hardness was observed for blends prepared with MDEHP.

Epoxy-based materials modified with melamine phosphates and zinc borate hydrate derivatives have potential applications in the electronics industry. For example, they can be used to make flame-retardant device housings, laminates, and insulating components where high thermal resistance and limited flammability are required. Such materials may find applications in the construction industry for protective coatings, structural adhesives, and blend panels to increase the resistance of components to high temperatures and fire.

## Figures and Tables

**Table 1:** Properties of melamine phosphate derivatives used as flame retardants.

Flame Retardant	Formula Mass (g/mol)	Nitrogen Content (mas% N)	Phosphate Content (mas% P_2_O_5_)	Zinc Content (mas% Zn)	Reaction Yield (mas%)
MP	224.12	37.48	31.68	–	97.0
MDBP	336.33	24.98	21.11	–	96.0
MBEHP	448.54	18.73	15.83	–	99.0
ZnB-MP	356.40	18.74	15.84	14.95	99.5

**Table 2:** Designations of blends and amounts of chemicals used for their preparations, as well as the contents of phosphate and phosphorus.

Sample	The Type of Flame Retardant	The Flame Retardant Content (mas%)	Amount (g)				Phosphate Content (mas% P_2_O_5_)	Phosphorus Content (mas% P)
			Epx601	IDA	TCPP	Flame Retardant		
Reference	–	–	10.00	5.00	1.00	–	–	–
MP_8	MP	8	10.00	5.00	1.00	1.28	2.53	1.10
MP_10	MP	10	10.00	5.00	1.00	1.60	3.17	1.38
MP_15	MP	15	10.00	5.00	1.00	2.40	4.75	2.07
MP_25	MP	25	10.00	5.00	1.00	4.00	7.92	3.46
MDBP_8	MDBP	8	10.00	5.00	1.00	1.28	1.69	0.74
MDBP_10	MDBP	10	10.00	5.00	1.00	1.60	2.11	0.92
MDBP_15	MDBP	15	10.00	5.00	1.00	2.40	3.17	1.38
MDBP_25	MDBP	25	10.00	5.00	1.00	4.00	5.28	2.31
MDEHP_8	MDEHP	8	10.00	5.00	1.00	1.28	1.27	0.55
MDEHP_10	MDEHP	10	10.00	5.00	1.00	1.60	1.58	0.69
MDEHP_15	MDEHP	15	10.00	5.00	1.00	2.40	2.37	1.03
MDEHP_25	MDEHP	25	10.00	5.00	1.00	4.00	3.96	1.73
ZnB-MP_8	ZnB-MP	8	10.00	5.00	1.00	1.28	1.27	0.55
ZnB-MP_10	ZnB-MP	10	10.00	5.00	1.00	1.60	1.58	0.69
ZnB-MP_15	ZnB-MP	15	10.00	5.00	1.00	2.40	2.38	1.03
ZnB-MP_25	ZnB-MP	25	10.00	5.00	1.00	4.00	3.96	1.73

**Table 3:** Glass transition temperatures (*T*_g_) and Shore hardness values obtained for the blends and reference sample.

Sample	*T*_g_ (°C)		Hardness D (°Sh)
	I ^a^	II ^b^	
Reference	54	78	76.0 ± 0.63
MP_8	47	61	71.0 ± 0.37
MP_10	52	68	66.1 ± 0.45
MP_15	42	65	72.0 ± 0.32
MP_25	26	43	70.3 ± 1.08
MDBP_8	43	58	72.0 ±0.55
MDBP_10	39	61	71.0 ± 0.71
MDBP_15	49	74	62.1 ± 0.37
MDBP_25	43	60	60.2 ± 0.68
MDEHP_8	38	55	69.1 ± 0.37
MDEHP_10	32	52	67.0 ± 0.32
MDEHP_15	45	52	58.6 ± 0.86
MDEHP_25	42	73	50.2 ± 0.75
ZnB-MP_8	49	62	73.0 ± 0.32
ZnB-MP_10	48	57	71.9 ± 0.80
ZnB-MP_15	38	60	73.2 ± 0.81
ZnB-MP_25	38	57	73.6 ± 0.58

^a^, ^b^ First and second heating cycle, respectively.

**Table 4:** TG-DTG data obtained for the blends and reference sample.

Sample	*T*_5%_ (°C)	*T*_10%_ (°C)	*T*_50%_ (°C)	*T*_max_ (°C)	Mass Losses (%)	Residual Mass (%)
Reference	141	184	362	164, 292, 356, 524	14, 12, 43, 29	1.6
MP_8	122	156	340	128, 289, 329, 534, 681	15, 21, 31, 27, 2	4.0
MP_25	127	158	327	137, 317, 521, 699	15, 52, 27, 3	3.0
MDBP_8	134	175	333	205, 334, 518, 689	15, 56, 24, 2	2.6
MDBP_25	140	182	336	172, 272, 336, 516, 705	12, 17, 45, 20, 3	2.8
MDEHP_8	129	164	336	142, 269, 339, 526, 712	13, 26, 32, 22, 4	2.9
MDEHP_25	123	157	322	132, 257, 328, 525, 698	12, 32, 27, 23, 5	3.1
ZnB-MP_8	167	214	341	229, 307, 331, 346, 516	14, 28, 8, 17, 28	4.7
ZnB-MP_25	143	188	360	155, 283, 344, 407, 535	13, 17, 28, 9, 21	12.2

**Table 5:** Flammability test results obtained using the PCFC method for the blends and the reference sample.

Sample	HRR_MAX_ (W/g)	THRR_MAX_ (s)	THR (kJ/g)	HRC (J/gK)
Reference	390	383	27.1	439
MP_10	275	337	25.8	313
MP_25	152	352	18.6	176
MDBP_10	188	341	23.2	221
MDBP_25	138	360	22.3	157
MDEHP_10	266	348	26.5	310
MDEHP_25	228	354	25.6	262
ZnB-MP_10	212	348	23.3	251
ZnB-MP_25	209	351	18.7	247

## Data Availability

The data supporting the findings of this study are available from the corresponding author upon reasonable request.

## Supplementary Materials

The following supporting information can be downloaded at: https://www.mdpi.com/article/10.3390/ma19132877/s1, Figure S1: ATR-FTIR spectra of pure epoxy resin and flame retardants.
