# Supplementary figures and images for: Epoxy Blends Containing Melamine Phosphate-Based Flame Retardants: Thermal and Flammability Performance
Source: Materials (Basel). 2026 Jul 5;19(13):2877. doi: 10.3390/ma19132877 (PMC13362991; doi:10.3390/ma19132877)

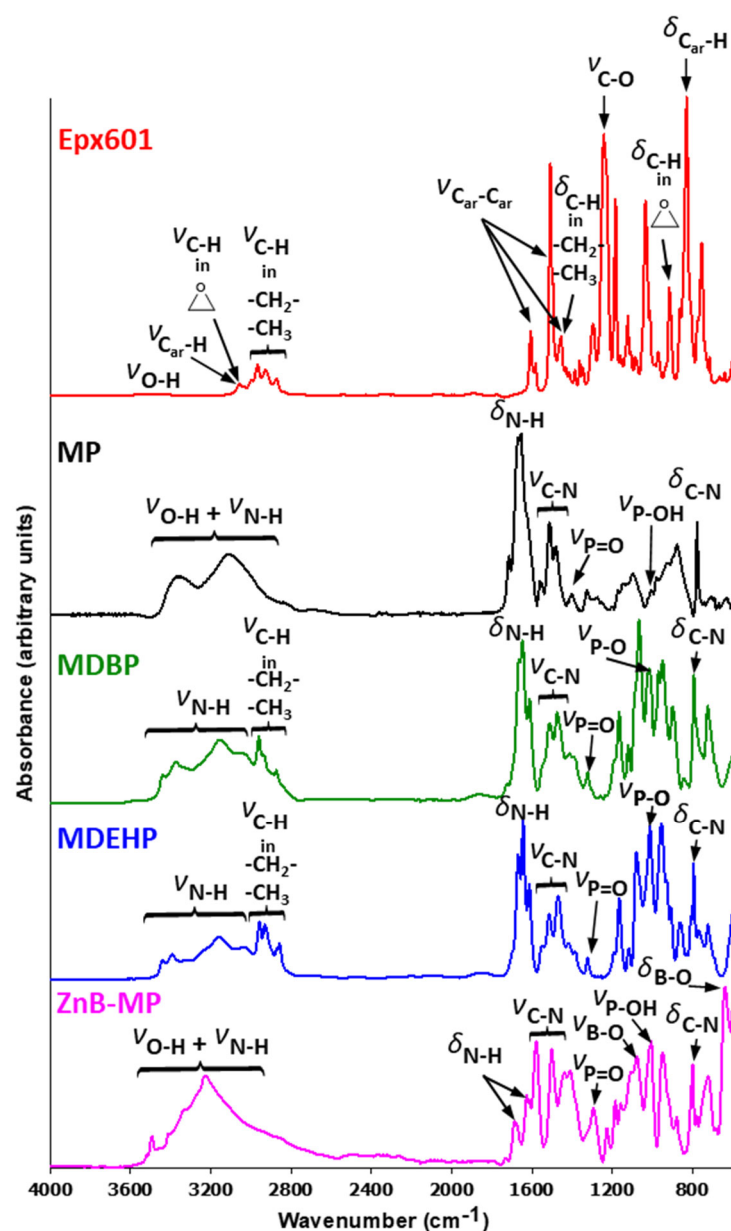

**Figure S1.** ATR-FTIR spectra of the pure epoxy resin and flame retardants.

Supplement: Supplementary file 1 [file materials-19-02877-s001.zip › materials-4361550-supplementary.pdf]
